# Supplementary material for: A genome-wide CRISPR screen defines host determinants of early Brucella infection in human macrophage-like cells
Source: Infect Immun. 2026 Jun 30;94(8):e00117-26. doi: 10.1128/iai.00117-26 (PMC13459713; doi:10.1128/iai.00117-26)
Supplement: Supplementary file 1 [file iai.00117-26-s0001.pdf]

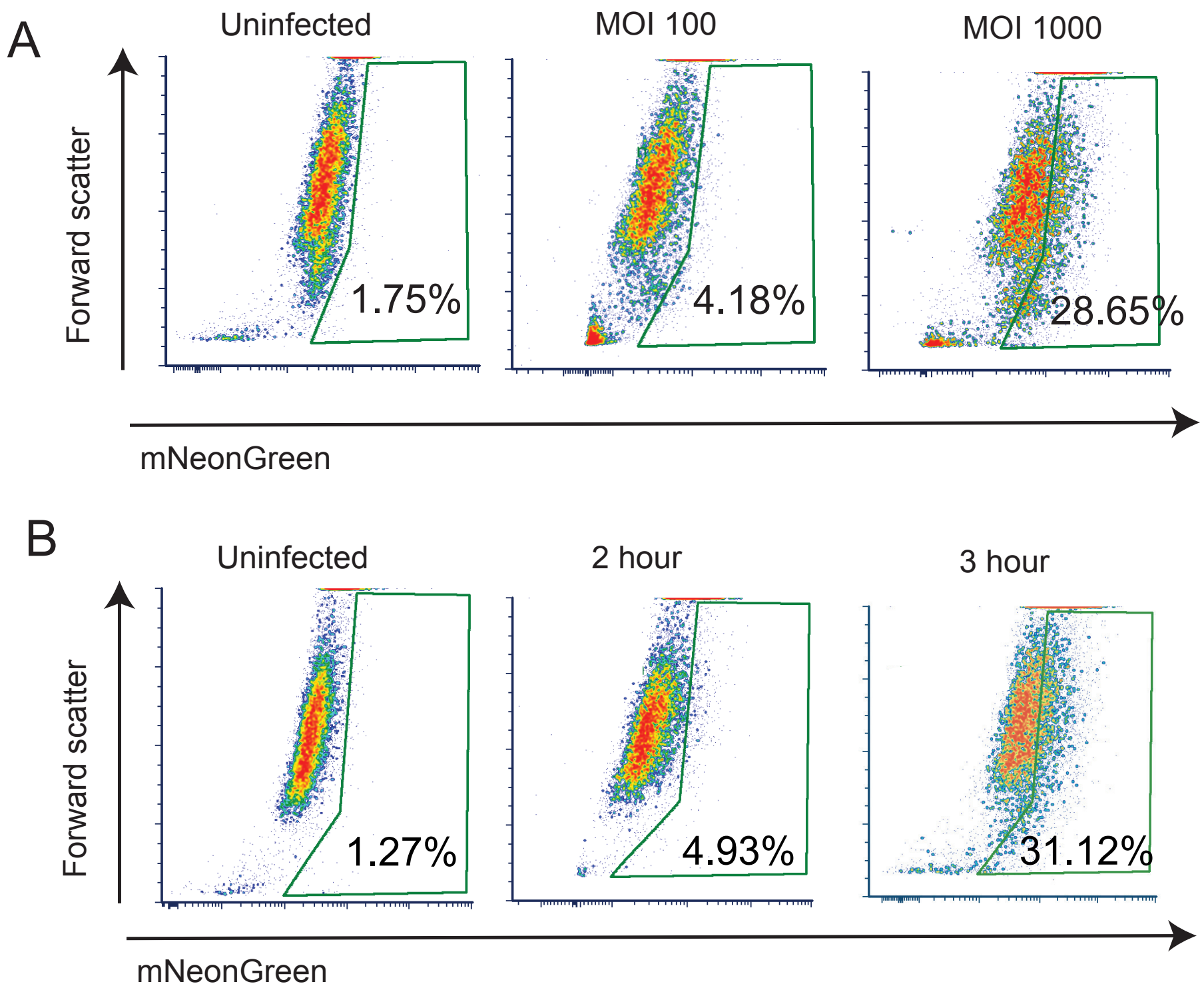

Figure S1. ***B. ovis*** **efficiently infects THP-1 macrophages at 3 hours post-infection at a multiplicity of infection (MOI) of 1,000.** (A) Representative flow cytometry plots showing mNeonGreen-positive THP-1 macrophages at 3 hours post-infection at increasing MOI. Plots are gated on live, single cells (gate shown as green box on flow plots) and the percentage of mNeonGreen-positive (infected) cells is indicated in each plot. (B) Representative flow cytometry plots showing mNeonGreen-positive macrophages at 2 and 3 hours post-infection with *B. ovis* at MOI 1,000. All plots are gated on live, single cells.

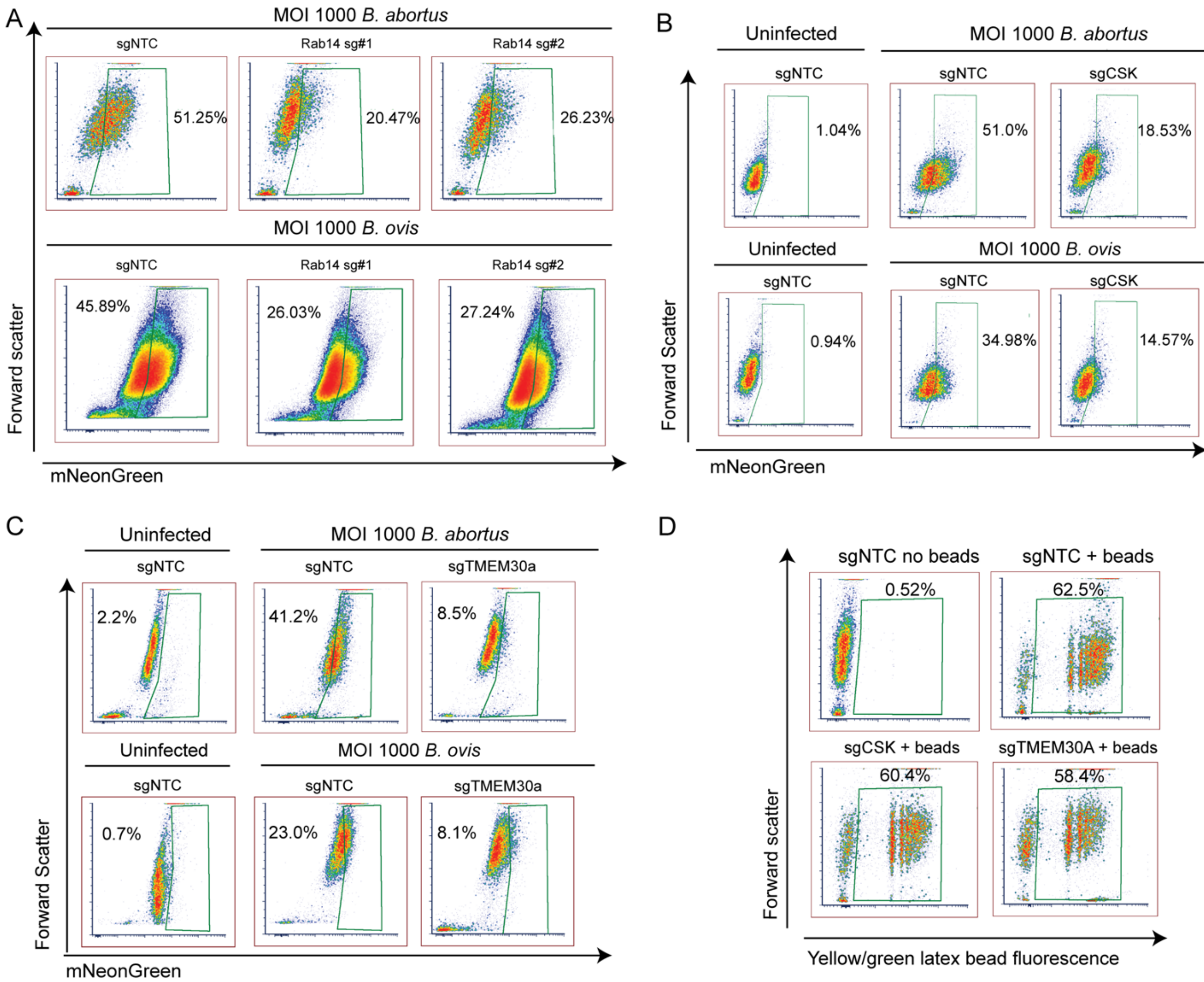

Figure S2. RAB14-, CSK-, and TMEM30A-deficient THP-1 macrophages are less susceptible to infection by *B. abortus* and *B. ovis*. THP-1 macrophages expressing non-targeting control (sgNTC) or the indicated sgRNAs were infected for 3 h with mNeonGreen-expressing *B. abortus* or *B. ovis* at MOI 1,000. Live, single cells were gated (gate shown as green box on flow plots), and the percentage of mNeonGreen-positive (infected) cells is indicated in each plot. (A) Representative plots for sgNTC cells and two independent RAB14-targeting sgRNA lines infected with *B. abortus* (top row) or *B. ovis* (bottom row). (B) sgNTC and CSK-deficient (sgCSK) cells, shown uninfected (left panels) or infected with *B. abortus* (top row) or *B. ovis* (bottom row). (C) sgNTC and TMEM30A-deficient (sgTMEM30A) cells, with uninfected sgNTC controls (left panels) and infections with *B. abortus* (top row) or *B. ovis* (bottom row). (D) Latex bead uptake in sgNTC, CSK-deficient, and TMEM30A-deficient macrophages. Cells were incubated with yellow-green fluorescent beads (bead:cell ratio 10:1) for 3 h. Representative plots show similar proportions of bead-positive cells across genotypes, indicating that phagocytic capacity is preserved in the mutant THP-1 lines.

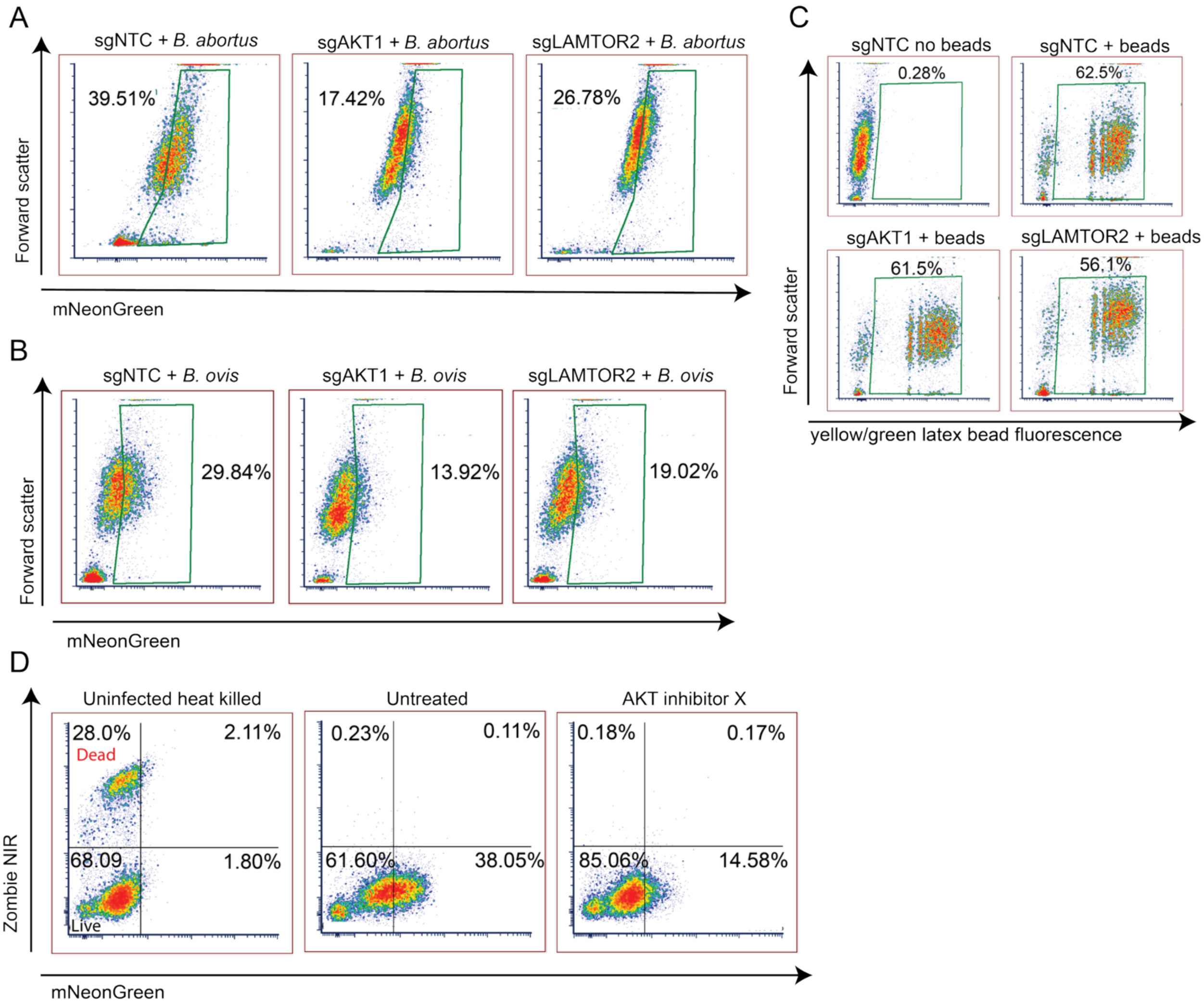

Figure S3. AKT1- and LAMTOR2-deficient THP-1 macrophages are less susceptible to infection by *B. abortus* and *B. ovis*, and pharmacological AKT inhibition reduces *B. abortus* infection without inducing cytotoxicity. THP-1 macrophages expressing non-targeting control (sgNTC) or sgRNAs targeting AKT1 or LAMTOR2 were infected with mNeonGreen-expressing *B. abortus* or *B. ovis* (MOI = 1,000) and analyzed 3 h post-infection by flow cytometry. (A, B) Representative flow cytometry plots showing the percentage of infected (mNeonGreen-positive) cells within the live, single-cell gate for each genotype after infection with *B. abortus* (A) or *B. ovis* (B). (C) Latex bead uptake in sgNTC, AKT1-deficient, and LAMTOR2-deficient macrophages. Cells were incubated with yellow-green fluorescent beads (bead:cell ratio 10:1) for 3 h. Representative plots show similar proportions of bead-positive cells across genotypes, indicating that phagocytic capacity is preserved in the mutant THP-1 lines. (D) Representative viability and infection profiles of THP-1 macrophages 3 h after infection with mNeonGreen *B. abortus* under the indicated conditions. Cells were gated (horizonal and vetical lines on the flow plot) on live, single events and stained with Zombie NIR viability dye. The left panel shows uninfected, heat-treated macrophages used to define live/dead gates. The middle and right panels show macrophages that were either untreated or pre-treated with 10 micromolar AKT inhibitor X prior to infection. Quadrants indicate the percentages of live/dead (Zombie NIR) and infected (mNeonGreen-positive) cells.

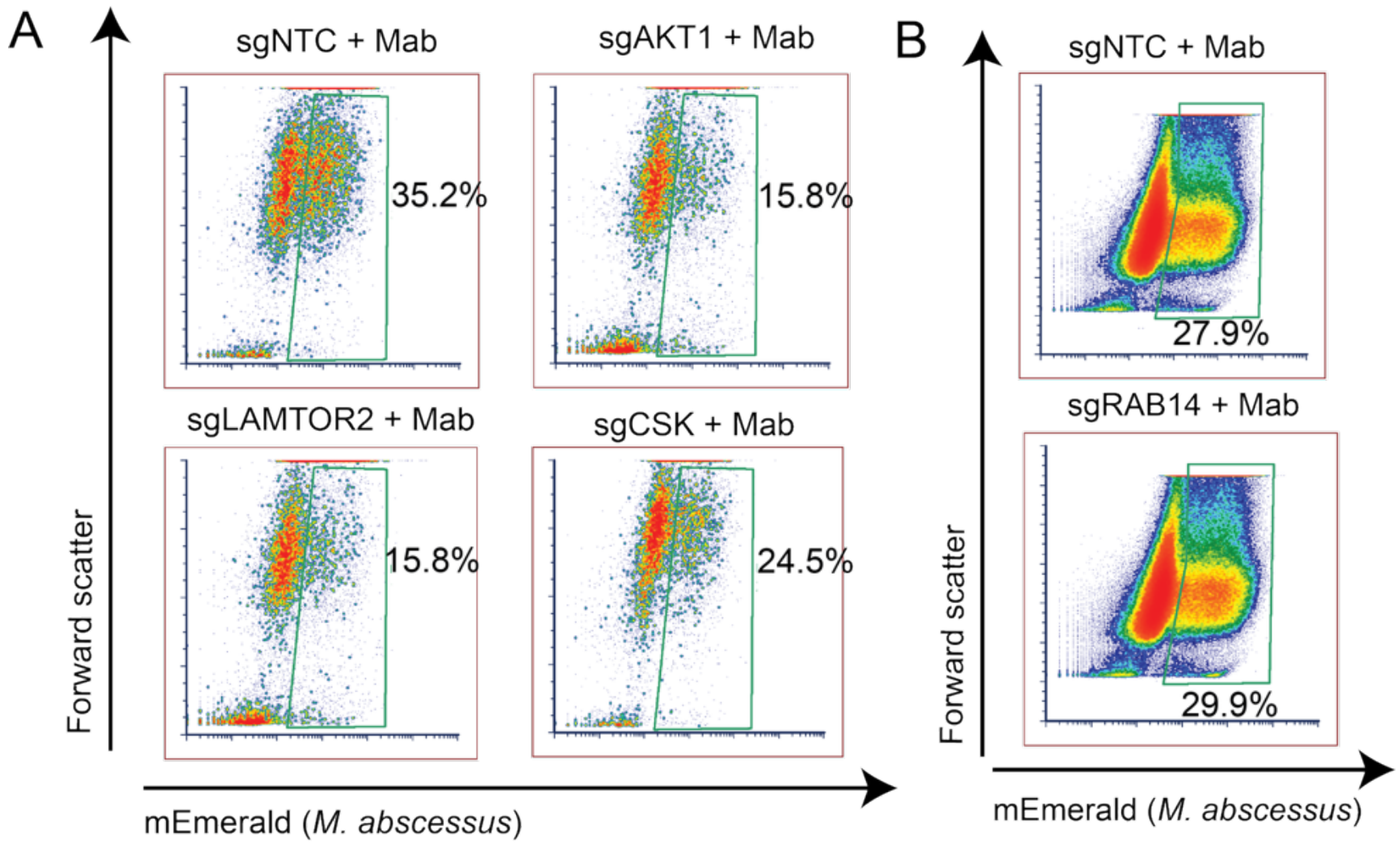

Figure S4. *Brucella* host factors also support *M. abscessus* infection, with the exception of RAB14. (A) Flow cytometry plots showing THP-1 macrophages expressing non-targeting control (sgNTC) or sgRNAs targeting AKT1, LAMTOR2, or CSK, infected with *mNeonEmerald*-expressing *Mycobacterium abscessus* at MOI = 5 for 4 hours. The percentage of mEmerald-positive (infected) cells is indicated for each genotype within the live, single-cell gate (gate shown as green box in the flow plots). (B) Representative flow cytometry plots for THP-1 cells expressing sgNTC or sgRNAs targeting RAB14, infected with *M. abscessus* under the same conditions. Unlike other validated Brucella host factors, disruption of RAB14 did not reduce *M. abscessus* infection, indicating a Brucella-specific requirement for this gene.

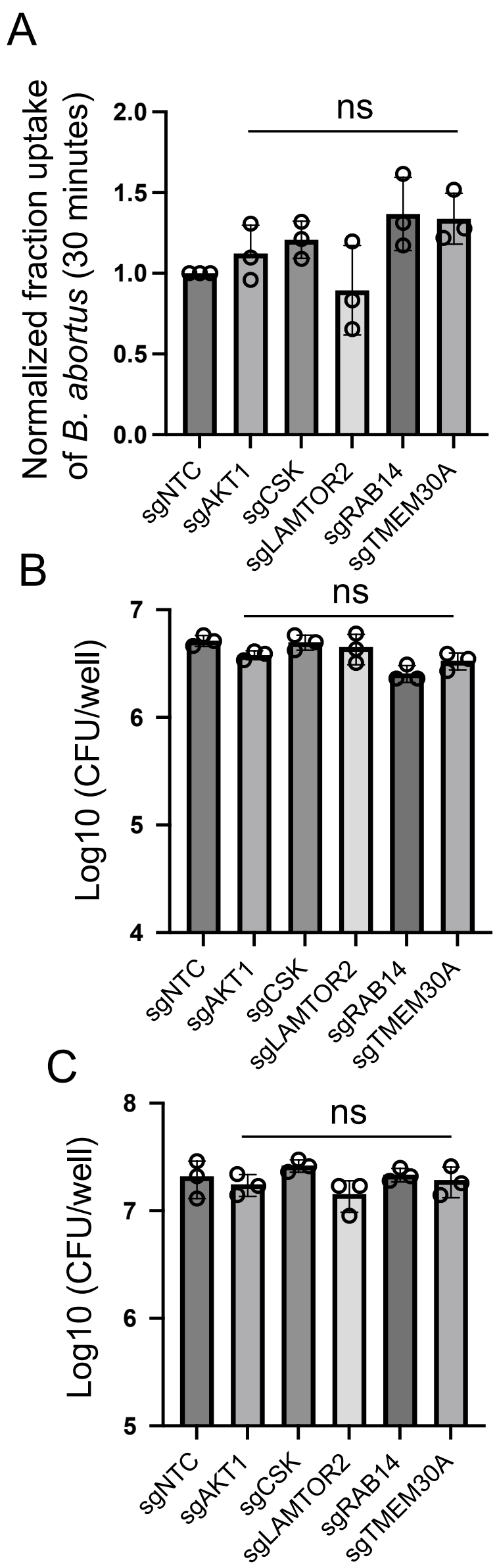

Figure S5. Validated host factor mutations do not impair *B. abortus* uptake or reduce viable intracellular burden as measured by gentamicin protection assay. THP-1 macrophages expressing a non-targeting control sgRNA (sgNTC) or sgRNAs targeting AKT1, CSK, LAMTOR2, RAB14, or TMEM30A were infected with *B. abortus*::mNeonGreen at MOI 1,000. (A) Bacterial uptake at 30 min post-infection quantified by flow cytometry and expressed as the fraction of mNeonGreen-positive macrophages normalized to sgNTC (set to 1.0). (not significant (ns); One-way ANOVA, Dunnett’s multiple comparison test to sgNTC) (B, C) Viable intracellular bacteria enumerated by gentamicin-protection assay at (B) 30 min and (C) 3 h post-infection, plotted as $\log_{10}$ CFU per well. Data represent mean ± SD from three independent biological replicates; open circles show individual replicate values. ns, not significant versus sgNTC by two-way ANOVA with Dunnett's multiple-comparisons test ($P > 0.05$ for all).
